# Salicylic Acid in Lumbago

**Published:** 1878-05

**Authors:** 


Salicylic Acid in Lumbago.—As this comparatively late addition to materia medica has been so much vaunted in the treatment of acute rheumatism, I think it will not be out of place to give my experience, though short, in its use in lumbago.

Notwithstanding lumbago is one form of chronic rheumatism, I have never seen salicylic acid recommended especially as its cure; but having been called during the month of July, 1877, and not knowing any specific, I determined to administer this acid and give it a fair trial. I shall describe the case below.

Since salicylic acid has been discussed so in its relation to acute rheumatlsm, I shall not stop to add my mite of experience in that direction, except that I have come to regard it as nearly a specific in that disease.

Case I.—Mr. D. D., aged forty-two, subject to lumbago for the last seven years, attacks occurring from five to eight times a year. I found him motionless, in bed; pain so intense on the slightest movement that he could not even flex his toes without excruciating pain. I gave him a No. 1 capsule (about ten or twelve grains), charged with salicylic acid, every two hours. After ten hours he could turn himself in bed; after twenty-four hours he got out of bed and walked to the dining-table in the next room; and after forty-eight hours he was out attending to his farm duties, with no pain when he walked carefully. When ten doses had been taken I ordered the capsule only four times a day, and advised him to rest a week and take nourishing diet. He told me his previous attacks had always lasted from one to three weeks; and said he: "This is the most severe attack I have ever had but one, and that lasted me three weeks instead of three days." He has had symptoms of attacks twice since; but he began at once on his capsules, and the symptoms passed away. He has gone longer this time free from lumbago than ever before during seven years.

Case II.—Mrs. M. H., aged thirty-two. Found her helpless, in bed, with no pain especially, except when she attempted to rise; could not walk without support on account of the severe pain in the lumbar region. I left her twelve capsules, same as in Case I, to be taken every three hours; sent her eight more capsules the next day; and had the satisfaction of learning from her husband, on the fourth day, that she was doing her household duties.

Case III.—Miss M. M., aged twenty; her second attack of lumbago. Complained of constant pain in the lumbar region; could only rest when the muscles of her back were on the stretch. Gave her ten capsules, to be taken every three hours; sent her ten more the next day; and met her riding to town, on horseback, on the fifth day after her first attack.

Case IV.—Mr. W. A., aged thirty-seven, first attack, came to my office and said he had been suffering for four days with severe pains in the muscles of his back, and also complained of pains and cramps in the flexor muscles of his thighs, and even lower down, especially the gastrocnemium. I gave him salicylic acid, always in capsules, about forty grains a day, for four days. Upon the sixth day he reported himself as having improved from the time he began his capsules.

In Case III there was a recurrence of the attack two months afterward. She sent immediately after feeling the first symptoms. I sent her six doses, to be taken every two hours. When I heard from her again she was well. I have never had unpleasant stomach symptoms from such large doses but once, and then by ceasing the acid a short time the symptoms disappeared. Never having read of lumbago succumbing so readily to treatment, I trust that the report ef these cases may not be without interest to your readers.—*Louisville Medical News.*

